# Imaging of cardiac fibroblast activation in a patient after acute myocardial infarction using ^68^Ga-FAPI-04

**DOI:** 10.1007/s12350-021-02603-z

**Published:** 2021-04-15

**Authors:** Susan Notohamiprodjo, Stephan G. Nekolla, Stephanie Robu, Alberto Villagran Asiares, Christian Kupatt, Tareq Ibrahim, Karl-Ludwig Laugwitz, Marcus R. Makowski, Markus Schwaiger, Wolfgang A. Weber, Zohreh Varasteh

**Affiliations:** 1 Department of Nuclear Medicine, Klinikum rechts der Isar, Technical University of Munich, 81675 Munich, Germany; 2 DZHK (German Centre for Cardiovascular Research), Partner Site Munich Heart Alliance, Munich, Germany; 3 1. Med. Klinik, Klinikum rechts der Isar, Technical University of Munich, Munich, Germany; 4 Department of Diagnostic and Interventional Radiology, Klinikum rechts der Isar, Technical University of Munich, Munich, Germany

**Keywords:** CAD, myocardial ischemia and infarction, PET, hybrid imaging, diagnostic and prognostic application, molecular imaging agents

## Abstract

Our previous study has demonstrated the feasibility of noninvasive imaging of fibroblast activation protein (FAP)-expression after myocardial infarction (MI) in MI-territory in a rat model with ^68^Ga-FAPI-04-PET. In the current extended clinical case, we sought to delineate cardiac uptake of ^68^Ga-FAPI-04 in a patient after MI with clinical indication for the evidence of fibroblast activation. Carcinoma patients without cardiac disease underwent ^68^Ga-FAPI-04-PET/CT as control. The patient with one-vessel disease underwent dynamic ^68^Ga-FAPI-04-cardiac-PET/CMR for 60 minutes. Correlation of cardiac ^68^Ga-FAPI-04 uptake with clinical findings, ECG, echocardiography, coronary-arteriography and enhanced cardiac-MRI with T1 MOLLI and ECV mapping were performed. No uptake was found in normal myocardium and in mature scar. A focal intense ^68^Ga-FAPI-04 uptake with continuous wash-out in the infarct territory of coronary occlusion correlating with T1 and ECV mapping was observed. The uptake of ^68^Ga-FAPI-04 extends beyond the actual infarcted area and overestimates the infarct size as confirmed by follow-up CMR.

## Introduction

Following myocardial infarction (MI), an orchestrated inflammatory response and reparative pathways are initiated, aiming to produce a robust and collagen-rich scar.^1^,^2^ The phenotype conversion of cardiac fibroblasts to their overly active counterparts, myofibroblasts, is the critical event in cardiac remodeling.^3^,^4^ Post-MI, a high abundance of extracellular matrix proteins is synthesized by myofibroblasts to replace myocyte loss and form a reparative scar. A balanced turnover of extracellular matrix via extracellular matrix synthesis by activated fibroblasts and degradation by matrix metalloproteinases is crucial for proper scar formation.^4^ The excessively increased activity of the fibroblasts resulting in excessive fibrosis within the myocardium is associated with poor patient prognosis. A diagnostic strategy targeted at detecting active ongoing fibrosis may provide critical insights into pathogenesis of heart failure. In addition, early detection of cardiac remodeling and fibrosis may be essential to prevent development of apparent heart failure.

The serine protease fibroblast activation protein (FAP) is a membrane-anchored enzyme which is specifically expressed in fibroblasts activated to differentiate to (proto-)myofibroblasts, but not in dormant fibroblasts or mature fibrocytes.^5^^68^Gallium-labeled FAP-inhibitor (FAPI) compound 04 (^68^Ga-FAPI-04) was initially introduced for PET imaging of cancer-associated fibroblasts.^6^ In our previous work, we demonstrated that image derived FAP expression after MI in a rat model allowed noninvasive PET imaging of activated fibroblasts.^7^

Further retrospective evaluations of cardiac FAPI distribution in a heterogeneous patient population with metastasized cancer associated with preexisting coronary artery disease, cardiovascular risk factors or metabolic disease were reported.^8^,^9^ However, ^68^Ga-FAPI-04-PET investigations in patients after MI have not been reported yet. For this case presentation, we retrospectively evaluated fibroblast activation in a patient after MI und carcinoma patients without cardiac disease.

## Case Presentation

All examinations had clinical indications and complied to the conditions of the updated Declaration of Helsinki (Section 37, unproven interventions in clinical practice) and the German Pharmaceutical Law (Section 13, 2b). In the controls, the indications for ^68^Ga-FAPI-04-PET/CT was the possible compassionate use of ^177^Lu-FAPI-radiotherapy^10^ and staging. In a patient after MI, the indications for ^68^Ga-FAPI-04-PET/MR was the compassionate use for chimeric antigen receptor T-cell-therapy^11^ of myocardial fibrosis and clarification of inflammation and viability after MI. Informed written consent for the investigation and scientific analyses were achieved in all patients.

### Finding in Patients with No History of Cardiac Diseases (Control)

**Figure 1:**
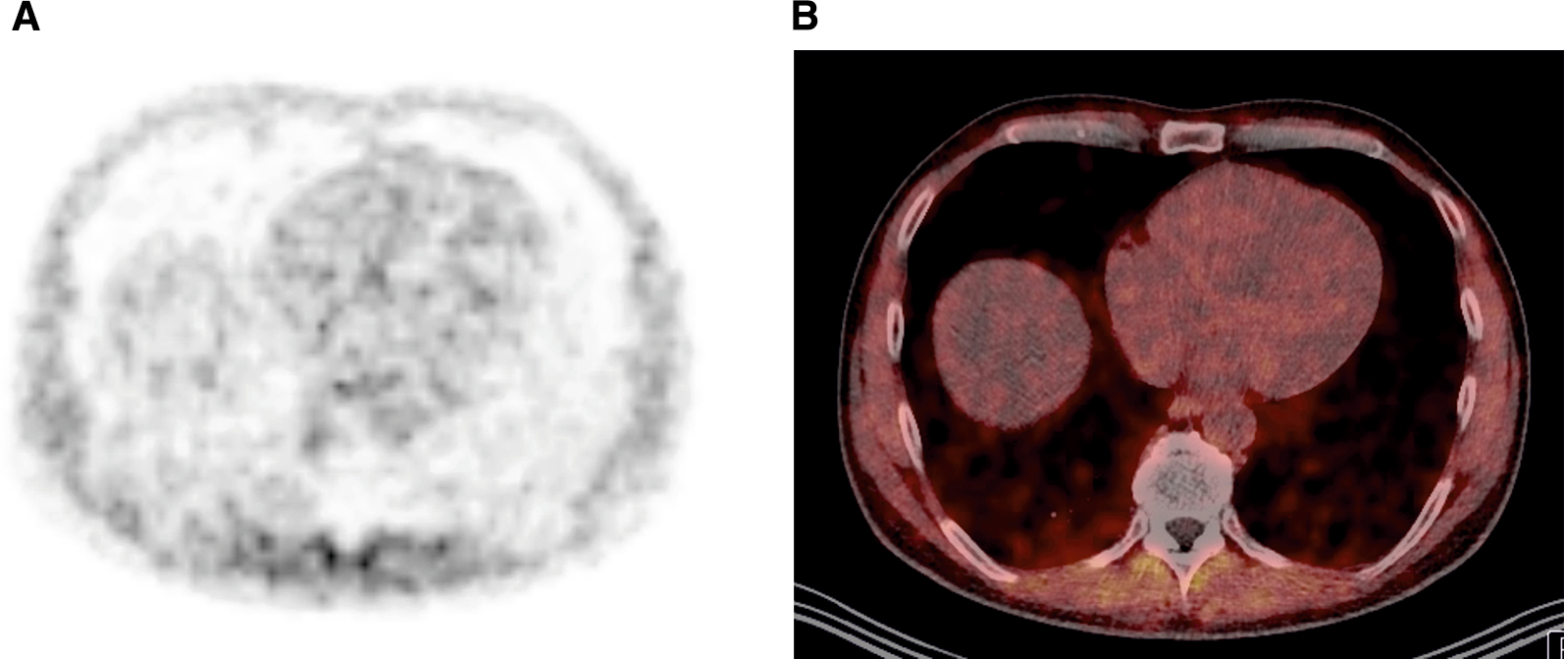
^68^Ga-FAPI-04 PET/CT Control patient. Representative reference patient without cardiac disease. No intense ^68^Ga-FAPI-04 uptake in normal myocardium was registered. **A** Attenuation corrected axial ^68^Ga-FAPI-04 PET. **B** PET/CT image fusion

Patient population of control group is summarized in Table 1. Normal myocardium showed activity uptake of ^68^Ga-FAPI-04 of similar intensity as blood pool activity indicating no specific uptake (Figure 1). The averages of maximum and mean standard uptake values (SUV_max_, SUV_mean_) are summarized in Table 2A.

**Table 1:** Patient population of control group

Population of control group	
Number	4
Sex	2 Males, 2 females
Age	Average 48 years (range 37-61 years)
Diagnosis	Metastasized carcinoma of osteosarcoma, breast cancer, tongue carcinoma, oropharynx carcinoma

**Table 2:** The standard uptake values (SUV) assessed in control patients and in the patient after STEMI

**A**	Control patients without cardiac disease	Average SUV_max_ ± standard deviation	Average SUV_mean_ ± standard deviation
	Myocardium (black)	1.2 ± 0.1	0.8 ± 0.1
	Blood pool (red)	1.5 ± 0.3	1.1 ± 0.4

**B**	Patient after STEMI	SUV_max_	SUV_mean_
	Injured area in LAD territory (black)	10.3	5.9
	Subendomyocardial area (green)	10.3	8.3
	Remote area (blue)	1.0	0.8
	Blood pool (red)	1.4	1.1

### Finding in Patient After Myocardial Infarction

A 33-year-old male was referred to intensive cardiac care unit due to acute STEMI followed by ventricular fibrillation. Return of spontaneous circulation after defibrillation. The present clinical findings at admission are summarized in Table 3. His coronary angiography showed severe sub-occlusive stenosis of medial LAD. Immediately, percutaneous coronary intervention and insertion of one drug eluting stent was performed. The pre- and post-interventional electrocardiograms are summarized in Table 4. Transthoracic echocardiography 5 days after MI showed normal left ventricular diameters, moderate myocardial thickening (interventricular septum 1.3 cm, posterior wall 0.9 cm), normal ventricular wall motion, but moderate left ventricular ejection fraction (LVEF) of 50%. CRP was persistently elevated at 80 mg·L^−1^. The patient complained about persistent dyspnea and fatigue. To investigate the cardiac fibroblast activation, a dynamic PET/MR imaging was performed 6 days after STEMI immediately after intravenous injection of 165 MBq ^68^Ga-FAPI-04.

**Table 3:** Present clinical findings of patient after STEMI at time of admission January 2020

Present clinical findings	
Symptoms	Acute nocturnal excruciating retrosternal pain, dyspnea, fatigue
Risk factors	Nicotine abuse, familial predisposition of CAD, no previous history of CAD
ECG	ST-elevation V1-V4
Troponin T	1270 ng·mL^−1^ (norm <0.014 ng/mL^−1^)
CK	944 U·L^−1^ (norm <174 U·L^−1^)
CK-MB	88 U·L^−1^ (norm 3-5% of total CK)
CRP	80 mg·L^−1^ (norm <5 mg·L^−1^)

**Table 4:** The findings of pre- and post-interventional ECG of patient after STEMI

	Pre-interventional	Post-interventional
Rhythm	Sinus	Sinus
Heart rate	58/s	61/s
Axis	Vertical	Indifferent
PQ and QT interval	Normal	Normal
R progression	Normal	Normal
ST segment	Horizontal elevation V1-3	No alteration of repolarization

**Figure 2:**
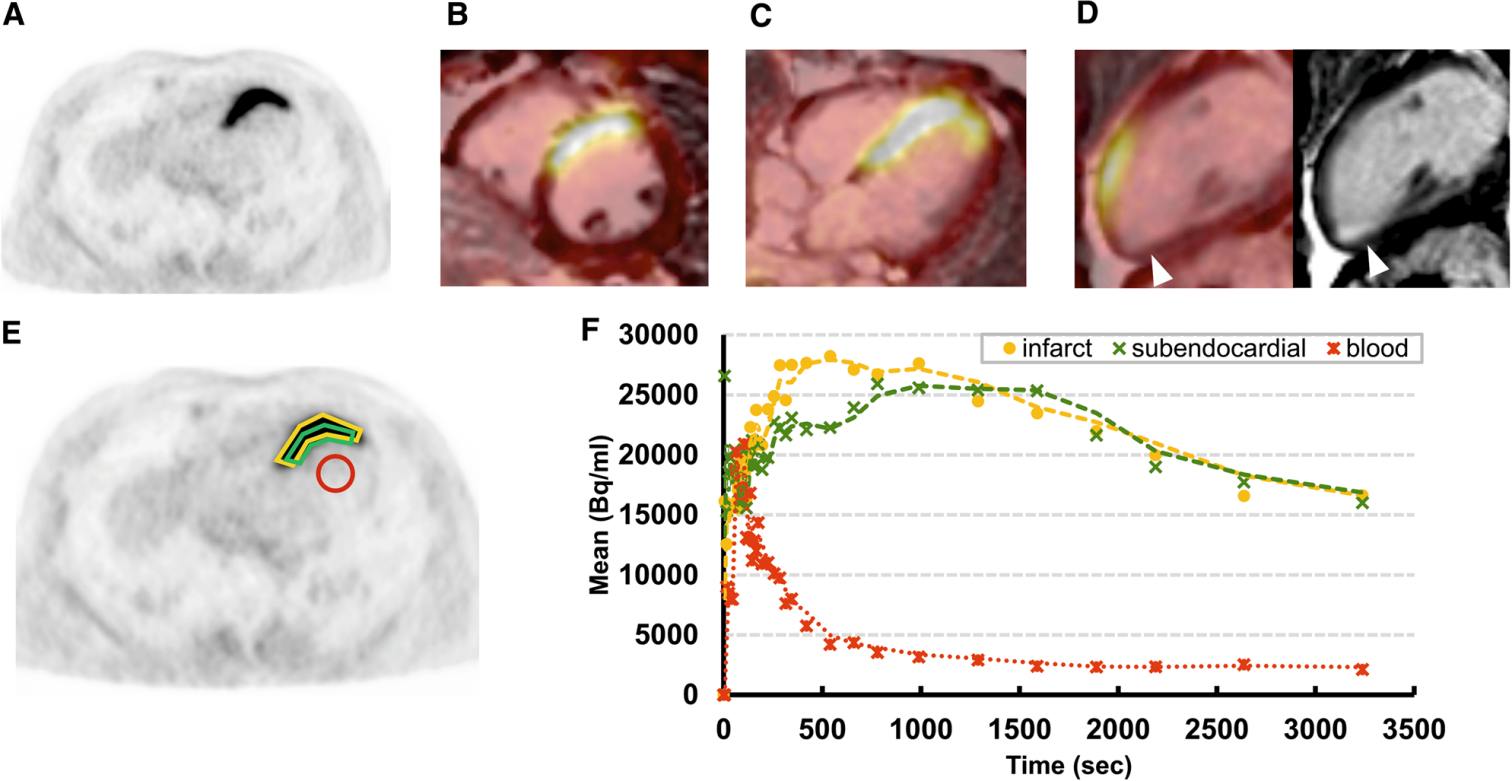
^68^Ga-FAPI-04 PET/CMR in a patient after MI. ^68^Ga-FAPI-04 PET/CMR in a patient after acute STEMI in LAD territory and ^68^Ga-FAPI-04 tracer kinetics. **A** Attenuation corrected axial PET. Fusion images of PET with 15 min late gadolinium enhancement sequences in **B** short axis, **C** horizontal long axis and **D** vertical long axis and corresponding MR. Arrowhead indicates small mature scar. **E** Example placement of ROI for dynamic analysis. **F** ^68^Ga-FAPI-04 tracer kinetics. Dots represent measured data, lines represent interpolation. Intense ^68^Ga-FAPI-04 uptake was observed in anterior and anterior septum wall in LAD territory. No significant ^68^Ga-FAPI-04 uptake is shown in the remote area similar as blood pool

^68^Ga-FAPI-04-PET of this patient showed focal intense uptake in the anterior and anterior-septal wall, which correlated well with the sub-occluded LAD territory (Figures 2A-D). Almost no uptake was registered in the remote remaining left ventricular wall with activity similar to blood pool. The assessed SUVs are summarized in Table 2B. A small mature scar in inferior apex (arrowhead in Figure 2D in CMR) showed almost no corresponding uptake. Tracer kinetics revealed after rapid peak accumulation a continuous wash-out of the activity in the anterior wall and in its subendocardial border zone (Figure 2F).

**Figure 3:**
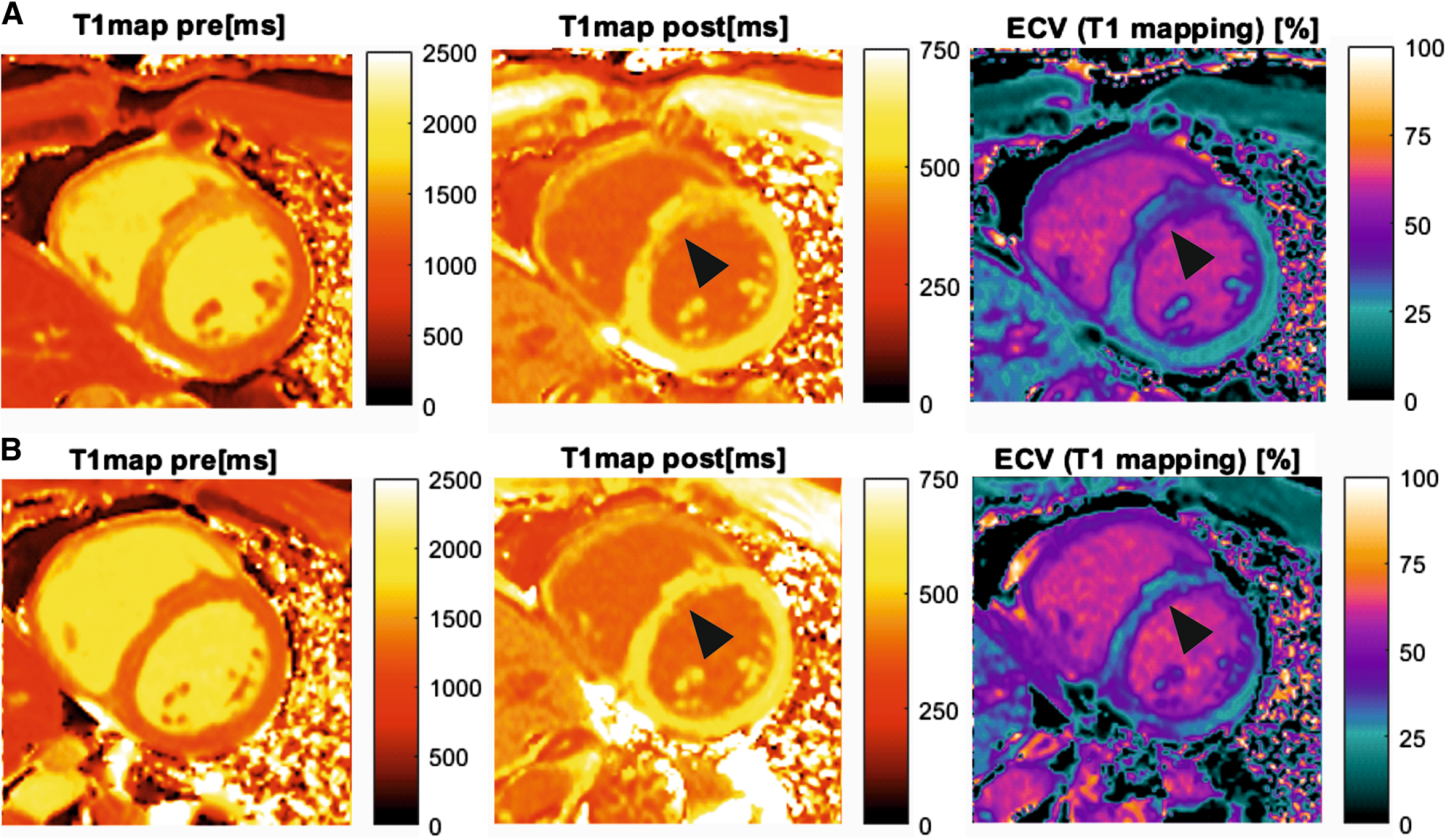
CMR of patient after MI. CMR of the patient with acute MI. **A** T1- and ECV mapping performed during simultaneous PET/CMR examination following STEMI in LAD territory. The focal intense myocardial ^68^Ga-FAPI-04 uptake (Figure 2B) is concordant with the alteration in T1 and ECV mapping. **B** Control CMR of the same patient after six months. T1- and ECV mapping showed regressive edema of the myocardium in the previously infarcted area and improving T1 and ECV parameters. A small subendocardial scar remains in the (antero-)septal wall in a similar extent as the distinct areas of decreased post-contrast T1-times and increased ECV (arrowhead)

The corresponding cardiac magnetic resonance (CMR) revealed in cine sequences a myocardial thickening of the anterior septum (14 mm end-diastolic) and thinning of the inferior apex with hypokinesia. The left ventricular function was moderately reduced (46%). In T2 weighted imaging, the anterior wall showed increased signal, suggesting myocardial edema. In dynamic myocardial perfusion imaging moderate hyperemia of the anterior wall could be observed. Early gadolinium enhancement sequences demonstrated a transmural enhancement of the anterior wall and adjacent septal segments, while late gadolinium enhancement revealed a sub-endocardial enhancement anterior-septal, but also a sub-endocardial enhancement in inferior apex. In T1 Modified Look-Locker Inversion Recovery (MOLLI) (Figure 3A) the anterior wall and adjacent septal segments showed in pre- and post-contrast enhancement pathological T1-relaxation-time and extracellular volume (ECV)-fraction in contrast to remote posterior-basal segments (Table 5). The corresponding ^68^Ga-FAPI-04-PET/CMR image fusion of the infarcted myocardium presented in Figures 2B-D showed a good correlation of the extent of CMR findings and ^68^Ga-FAPI-04 intense uptake with the LAD territory. But the extent of intense ^68^Ga-FAPI-04 uptake was slightly larger than the extent of the pathological CMR findings.

**Table 5:** T1 MOLLI and ECV of the patient after STEMI and 6 months follow-up

Hematocrit = 0.4	6 days after STEMI			6 months follow-up		
	T1 pre (ms)	T1 post (ms)	ECV (%)	T1 pre (ms)	T1 post (ms)	ECV (%)
Injured area in LAD territory	1,589	368	57	1,388	372	55
Adjacent myocardium	1,398	517	33	1,264	569	27
Remote myocardium	1,197	584	24	1,257	570	27
Skeletal muscle	1,179	692		1,137	782	
Liver	796	406		794	416	
Blood pool	1,906	368		1,991	379	

Control CMR 6 months after MI revealed discrete sub-endocardial late enhancement in the inferior apex and anterior-septal wall, indicating scar tissue which is significantly smaller than the extent in the sub-acute of ^68^Ga-FAPI-04 scan. The left ventricular function recovered to almost normal with LVEF 60%. No hypokinetic ventricular wall motion was detected, except a discrete hypokinesia in the apex. Apart from the anterior-septal sub-endocardial scar (arrowhead in Figure 3B), the assessed pre- and post-contrast T1 MOLLI and ECV (Figure 3B) showed normalization of the findings (Table 5).

## Discussion

In the present retrospective analysis of cardiac ^68^Ga-FAPI-04 uptake in patients with no prior history of cardiac disease and in a patient after acute MI, we found substantial different results (Figures 1A, 2A). In patients without history of cardiac disease, the myocardium showed a homogenous tracer uptake similar to the blood pool activity (Figure 1). Whereas, in the patient with acute MI, a focal intense uptake of ^68^Ga-FAPI-04 was registered in the infarct territory of the occluded coronary artery, indicating local enhanced fibroblasts activation. The tracer uptake in the remote myocardium (the myocardium appearing unremarkable in CMR) was similar to normal myocardium of the control group, indicating the absence of active fibroblasts in healthy myocardium^5^ (Figure 2; Table 2). A mature myocardial scar in inferior apex detected in CMR showed no uptake of ^68^Ga-FAPI-04 (arrowhead in Figure 2D), indicating no further FAP expression in established disease with fixed fibrosis because of significant reduction of activated fibroblast density during infarct maturation.^12^

A major determinant of post-MI remodeling severity is the infarct size. In the present study, we observed that the extent of ^68^Ga-FAPI-04 uptake in the infarcted area overestimates the actual infarct size. This finding is in line with recent data obtained from different animal studies showing increased fibroblast activity in adjacent MI (MI border zone).^7^,^13^ This finding indicates that the extent and the intensity of ^68^Ga-FAPI-04 uptake do not represent solely myocardial scaring process. But it also indicates more likely overlapping enhanced myocardial fibroblast migration in an inflammatory, proliferative process, such as in viable but ischemic jeopardized border zone or hibernating myocardium. The presence of active fibroblasts in the cardiac interstitium of the hibernating myocardium is reported to be an important indicator in determining recovery of function after revascularization.^14^ As confirmed by the control CMR investigation performed 6 months after MI, it is remarkable that the extent of the resulting scar is definitely smaller than in the first examination performed 6 days after MI.

Contrast-enhanced CMR offers high spatial resolution and can identify acute myocardial infarction and myocardial scar.^15^ CMR assessment and differentiation of pathological cellular, vascular and interstitial myocardial alterations with T1 and ECV mapping may be useful in the estimation of the degree of fibrosis or volume of myocardial collagen. The concordance of the focal intense myocardial ^68^Ga-FAPI-04 uptake with the alteration in T1 and ECV mapping registered in our patient is remarkable (Figures 2, 3). However, the wide range of reported correlations between T1 and ECV estimates and histo-morphological parameters, which vary from poor to excellent, limits the potential of CMR.^16^ More importantly, the measured ECV values reflect an indirect evidence of fibrosis. But other pathologies resulting in an expansion of extracellular space, such as inflammatory edema or protein deposition, can lead to increased ECV values. Therefore, CMR techniques seem not to be specific for fibrosis.^16^ Furthermore, these measurements tend to reflect a relatively late product of fibroblast activation. Noninvasive imaging of activated fibroblasts with ^68^Ga-FAPI-04-PET, however, may have a potential to specifically visualize fibrotic process already at its onset. It could therefore provide unique opportunities to study cardiac remodeling at molecular level over time and to monitor therapeutic interventions that aim to prevent a progressive decline of ventricular function.^17^ The simultaneous ^68^Ga-FAPI-04-PET/CMR may boost the diagnostic potential of ^68^Ga-FAPI-04-PET by additional information achieved from CMR in identification of early manifestation of remodeling amenable to preventive intervention. To confirm our preliminary results and to further investigate the comprehensive pathophysiology, further studies with a larger population are encouraged.

## Conclusion

As shown in this case of a patient after STEMI, the enhanced FAP activation in acutely injured myocardium was identified und visualized with ^68^Ga-FAPI-04-PET. Noninvasive assessment of activated fibroblasts may provide unique opportunities to identify early manifestation of cardiac remodeling amenable to preventive intervention.

## Abbreviations

CMR: Cardiac magnetic resonance
CT: Computed tomography
ECV: Extracellular volume fraction
FAP: Fibroblast activation protein
FAPI: Fibroblast activation protein inhibitor
LVEF: Left ventricular ejection fraction
MOLLI: Modified Look-Locker Inversion Recovery
MR: Magnetic resonance
SUV: Standard uptake value
